# Implication of the Polymeric Phenolic Fraction and Matrix Effect on the Antioxidant Activity, Bioaccessibility, and Bioavailability of Grape Stem Extracts

**DOI:** 10.3390/molecules28062461

**Published:** 2023-03-08

**Authors:** Juan Antonio Nieto, Irene Fernández-Jalao, María de las Nieves Siles-Sánchez, Susana Santoyo, Laura Jaime

**Affiliations:** Institute of Food Science Research (CIAL), Autonomous University of Madrid (Universidad Autónoma de Madrid (CEI UAM + CSIC)), 28049 Madrid, Spain

**Keywords:** grape stem, phenolic compounds, digestion, bioaccessibility, bioavailability, antioxidant activity, by-products, matrix effect

## Abstract

The bioaccessibility and bioavailability of phenolics compounds of two grape stem extracts with different composition were studied. High polymeric extract (HPE) presented a higher content of total phenolics (TPC), procyanidins, hemicelluloses, proteins, and ashes, whereas low procyanidin extract (LPE) showed a higher fat, soluble sugars, and individual phenolic compounds content. Corresponding to its higher total phenolics content, HPE possesses a higher antioxidant activity (TEAC value). The digestion process reduced the antioxidant activity of the HPE up to 69%, due to the decrease of TPC (75%) with a significant loss of polymeric compounds. LPE antioxidant activity was stable, and TPC decreased by only 13% during the digestion process. Moreover, a higher antioxidant phenolic compounds bioavailability was shown in LPE in contrast to HPE. This behaviour was ascribed mainly to the negative interaction of polymeric fractions and the positive interaction of lipids with phenolic compounds. Therefore, this study highlights the convenience of carrying out previous studies to identify the better extraction conditions of individual bioavailable phenolic compounds with antioxidant activity, along with those constituents that could increase their bioaccessibility and bioavailability, such as lipids, although the role played by other components, such as hemicelluloses, cannot be ruled out.

## 1. Introduction

Over the last few decades, winemaking by-products have been used to obtain high-value-added natural ingredients, which have several potential health benefits, attributed to the presence of certain phenolic compounds [1,2]. Several extraction techniques have been proposed for obtaining these compounds, including traditional solid–liquid extractions, as well as more efficient techniques, such as pressurized liquid extraction (PLE) [1,3].

Extracts from winemaking by-products are well known to possess antioxidant substances, generally linked to their total phenolic content, besides the specific antioxidant capacity shown by each phenolic compound [3,4]. Among the valorisation of these side streams, pomace and grape seeds have been extensively investigated as sources of natural antioxidants [5,6]. Despite this, grape stems, generated in large quantities in the wine industry, have received much less attention. In this respect, some research papers have highlighted the use of grape stems as an alternative source of antioxidants [3,7]. These antioxidant capacities are a consequence of their phenolic composition, mainly comprised of flavan-3-ol monomers (catechin and epicatechin), dimers (dimer B_1_ and dimer B_2_), and polymers, along with several phenolic acids (gallic acid and caftaric acid, among others), quercetin and quercetin derivatives, as well as stilbenes such as *trans*-resveratrol and *trans*-piceid [7,8].

However, when considering the potential health benefits of phenolic extracts, both bioaccessibility and bioavailability should be taken into account. Despite the antioxidant potential of extracts from winemaking by-products, some studies have shown that this capacity may be decreased during the digestion process because of a reduced bioaccessibility of various phenolic compounds [6,9,10]. Moreover, phenolic compounds are used to display low bioavailability, although there are remarkable differences between them, which affects the actual antioxidant activity of these extracts [11,12]. This bioavailability depends on several factors, including matrix composition, digestibility, stability, intestinal absorption, distribution, and metabolism [9,11]. In this context, it is well known that phenolic compounds can interact with carbohydrates, fibres, lipids, or proteins, among other food constituents [13,14,15]. However, there are scarce studies about the bioavailability of phenolic compounds of plant extracts as affected by other components, and, in particular, only a few studies have focused on the bioaccessibility and bioavailability of grape stem extracts and their effect on antioxidant activity. Moreover, these studies showed mixed results about the bioaccessibility and bioavailability of phenolic compounds [7,9,15,16]. Indeed, significant differences in the bioaccessibility of phenolic compounds of diverse grape by-product extracts have been reported [5,10]. This fact clearly supports the idea of the important influence of the matrix on the bioaccessibility of phenolic compounds during the digestion process, as Ferreyra et al. [9] suggested. Moreover, it is important to point out that scarce information of the possible role of the polymeric phenolic fraction of foods or plant extracts on the total phenolic bioaccessibility, bioavailability, and antioxidant activity during the digestion process can be found in the literature. Therefore, more studies are required. In this regard, to our knowledge, no studies have previously been conducted to elucidate the phenolic compound bioavailability of grape stem extracts and its potential antioxidant activity.

In this research, the bioaccessibility and the bioavailability of antioxidant phenolic compounds of grape stem extracts were investigated. For this purpose, two PLE stem extracts, with a very different composition and antioxidant activity, were submitted to an in vitro gastrointestinal digestion process followed by simulation of the intestinal absorption using a Caco-2 cell model, a widely used in vitro model for human intestinal drug absorption. The aim of the present study was to enhance the knowledge about the influence of the digestion process and intestinal absorption on antioxidant phenolic compounds from grape stems and the possible matrix effect.

## 2. Results

### 2.1. Characterization of PLE Extracts

Two PLE stem extracts with noticeably different composition and antioxidant activity were studied based on our previous research work [17]. As can be seen in Table 1, soluble sugars, protein, fat, ashes, phenolic compounds, and total carbohydrates were found in both extracts, except for carbohydrates, which were only found in the high polymeric extract (HPE). HPE was characterized by a significantly higher protein, ashes, total phenolics and procyanidins, a lower fat content, and a slightly lower amount of soluble sugars than the low polymeric extract (LPE). Moreover, a high TEAC value was found in HPE (0.998 ± 0.005 mmol Trolox/g extract), whereas LPE presented a moderate one (0.212 ± 0.010 mmol Trolox/g extract). Additionally, the TEAC value of the isolated mono-oligomeric fraction (obtained by an ultrafiltration process) from HPE was 0.148 ± 0.002 mmol Trolox/g extract, denoting the potential antioxidant activity of the fraction of polymeric procyanidins.

The phenolic composition analyses conducted by HPLC-PAD showed that both extracts mainly consisted of flavan-3-ols, in particular, catechin and, to a lesser extent, epicatechin. Procyanidin B_1_ was the most representative among dimers. Some phenolic acids—such as gallic, caftaric, vanillic, or syringic acids, as well as *trans*-resveratrol, quercetin, and quercetin derivatives—were also identified (Table 2). 

Beyond this common phenolic profile, quantitative differences were observed between both extracts. LPE showed slightly higher amounts of phenolic acids (except for caftaric and 3-coumaric acids), along with a greater content of catechin, stilbenes, and quercetin derivative compounds, compared to HPE. However, higher quantities of procyanidin dimers or quercetin-3-*O*-glucuronide were found in HPE compared to LPE.

Procyanidin characterization of HPE revealed a medium degree of polymerization (mDP) of 12 units. Structural composition determined epicatechin (72%) and catechin (15%) as the main structural units. Additionally, 12% of the total constituent units of procyanidins were galloylated. LPE procyanidin characterization revealed a similar composition (epicatechin, 67%, catechin 17%, among other minor flavan-3-ol units) and mDP (12 units), where 16% of the total constituent units of procyanidins were galloylated (Table S1).

### 2.2. In Vitro Gastrointestinal Digestion: Antioxidant Activity and TPC

**Figure 1:**
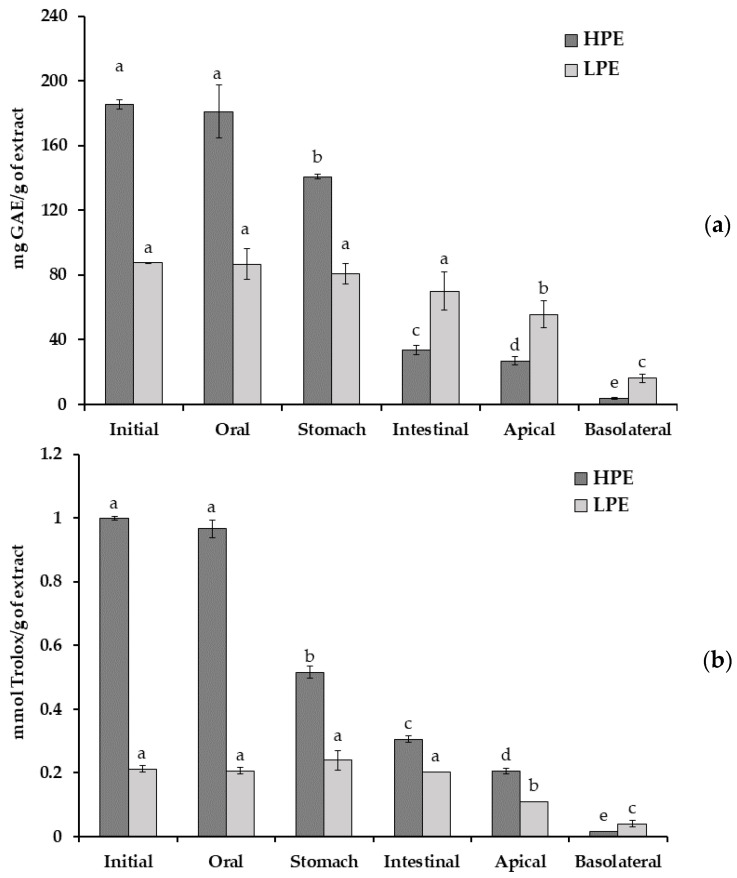
TPC (**a**) and antioxidant activity (**b**) of HPE and LPE during the simulated digestion and transepithelial transport on the Caco-2 absorption process. Data represent mean ± SD (*n* = 9). ^a–e^ Different letters indicate significant differences during the digestion process of each extract using the LSD test (*p* < 0.05).

The antioxidant activity of both extracts during the digestion process is shown in Figure 1. Oral digestion produced no significant changes in antioxidant activity of HPE, while stomach and intestinal steps resulted in important decreases. Thus, the antioxidant activity of HPE was reduced from 0.998 ± 0.005 mmol Trolox/g extract (undigested extract) to 0.305 ± 0.009 mmol Trolox/g extract after the digestion process (decreased 69%). However, the antioxidant activity of LPE was not modified across the digestion process.

Regardless of TPC, a similar trend was observed as total phenolics of HPE decreased sharply, while those of LPE decreased only slightly after the digestion process (Figure 1).

Changes in the antioxidant activity of the HPE during the digestion process were mainly associated with the polymeric fraction (r = 0.996; *p* < 0.001) compared to the monomeric and oligomeric compounds (r = 0.694; *p* < 0.05), suggesting a higher contribution of the polymeric fraction on the whole antioxidant activity during this physiological process (Figure S1).

### 2.3. In Vitro Gastrointestinal Digestion: Phenolic Compound Composition

Both extracts showed a similar trend during gastrointestinal digestion, characterized by a decrease in the phenolic content as the gastrointestinal digestion progressed (Table 3 and Table 4).

The oral phase showed a marginal effect on the phenolic composition, where only slight changes occurred in some specific compounds. Meanwhile, stomach and intestinal phases were critical steps in the bioaccessibility of both extracts. In general, the gastric phase affected the phenolic composition of both extracts similarly, with reductions up to 29%. However, a higher decrease in these compounds was observed for HPE, which caused a considerably lower bioaccessibility of phenolic compounds of HPE compared to LPE (29% and 52%, respectively). 

Phenolic acids were found to be more stable compounds compared to other phenolic constituents throughout the digestion process. Slight increases of 4-hydroxybenzoic acid and syringic acid were determined during the gastric step for both extracts, as well as for gallic acid in LPE. However, phenolic acid reductions were determined after intestinal digestion. HPE showed a lower bioaccessibility of hydroxybenzoic acids (55%) compared to LPE (71%), with significant reductions of gallic acid for HPE. Similar values were found for hydroxycinnamic acids (close to 71%).

Stilbenes were also relatively stable in the digestion process. LPE showed slight increases of *trans*-resveratrol, whereas in HPE, it was reduced approximately 24%. Regarding flavonols, a less-pronounced loss was observed after the digestion process, with a bioaccessibility of 78% in both extracts.

**Figure 2:**
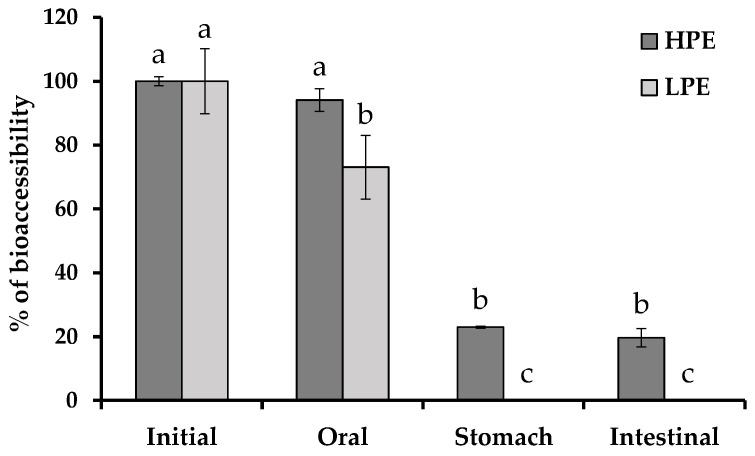
Bioaccessibility of the polymeric phenolic compounds of HPE during the simulated digestion process. Data represent mean ± SD (*n* = 9). ^a,b,c^ Different letters indicate significant differences between digestion steps in a group using the LSD test (*p* < 0.05).

Moreover, flavan-3-ols showed a significant decrease during the digestion process of both extracts, mainly after the intestinal step. Catechin (the major component of this group) was reduced 86% and 68% for HPE and LPE, respectively. Similar results were determined for epicatechin in HPE whereas its bioaccessibility in LPE was superior (50%). Conversely, a high amount of dimer B_1_ remained after gastrointestinal digestion (70%). Among flavan-3-ols, an intensive decrease of polymeric procyanidins occurred during the digestion process of HPE. Sixty-three percent of these compounds were lost during the gastric step, reaching a final bioaccessibility of 26% (Figure 2), and they were not detected after the stomach phase.

### 2.4. In Vitro Transepithelial Transport of Extracts

Transepithelial transport experiments of the digested extracts were carried out using an in vitro model of the intestinal barrier: Caco-2 cells differentiated to enterocytes [18]. Prior to transport experiments, the cytotoxicity of the digested extracts was evaluated at 6 h. The results showed that 100 µL of both digested extracts was the maximum volume that did not significantly affect cell viability (>99%). In addition, the integrity of the Caco-2 monolayer during exposure experiments was monitored as the TEER value.

Both digested extracts showed a lower antioxidant activity after their intestinal absorption. The TEAC value determined for the HPE basolateral chamber was 0.015 ± 0.000 mmol Trolox/g of extract, whereas the TEAC value of the LPE basolateral chamber was 0.040 ± 0.009 mmol Trolox/g of extract. Therefore, the antioxidant activity detected in the basolateral fraction of LPE was 2.6 times superior to the HPE basolateral fraction (Figure 1).

The TPC of the apical and basolateral fractions of both digested extracts was measured to determine the quantity of compounds that remained unabsorbed and bioavailable, respectively (Figure 1). The bioavailability was higher for LPE (23%) compared to HPE (11%). Thereby, the bioavailable fraction from LPE presented 16.304 ± 2.550 mg GAE/g extract while HPE showed 3.691 ± 0.432 mg GAE/g extract.

### 2.5. In Vitro Transepithelial Transport: Phenolic Compound Transport

Similar bioavailable phenolic compounds were observed in basolateral fractions of both extracts. They were primarily comprised of flavan-3-ols, phenolic acids, and stilbenes. Nevertheless, important differences were noticed between them (Table 5). The main component detected in the basolateral fraction of both extracts was catechin, where LPE presented a quantity three times superior compared to HPE. Nevertheless, epicatechin and dimer B_1_ amounts were determined only in the LPE basolateral fraction. 

Other phenolic compounds—consisting of phenolic acids, flavonols, and stilbenes—were also determined in the basolateral chamber. Diverse phenolic acids were found, being principally comprised of syringic and gallic acids for HPE, and vanillic and syringic acids for LPE. Both extracts presented similar amounts of quercetin-3-*O*-glucuronide. *Trans*-piceid and *trans*-resveratrol were also presented in the basolateral fraction of both extracts, but higher amounts were quantified in LPE.

## 3. Discussion

### 3.1. Characterization of PLE Extracts

The composition of both extracts was consistent with the main components found in Merlot’s grape stems [19]. A higher content of lipids and a slightly higher amount of soluble sugars—along with a lower extraction of proteins, minerals, and phenolic compounds—agreed with the lower polarity of ethanol (LPE solvent) compared to ethanol:water 30% (HPE solvent). The presence of other components of grape stems—such as cellulose, soluble, and insoluble acid lignin—was discarded in either of the two extracts, as liquid extracts were obtained. This result agrees with other studies, as these components of plant cell walls are known not to be soluble under the extraction conditions (solvents, temperature, and time) used in this study [20,21]. Moreover, the absence of hemicelluloses in LPE was confirmed as no ethanol insoluble residue was found in this extract. Total carbohydrates, obtained after precipitation of defatted HPE with absolute ethanol, were quantified on the basis of xylose, as xylans are proposed to be the main hemicelluloses present in grape stems [19]. 

The TPC and TEAC values determined for HPE and LPE were similar to those found in other PLE grape stem extracts [1,3]. The greater phenolic content observed for HPE was attributable to its higher polymeric procyanidin content, whereas LPE was mainly composed of mono-oligomeric phenolic compounds. Linked to this different content in polymeric procyanidins, a similar trend was determined for antioxidant activity, being superior in HPE. It is well known that the antioxidant activity of grape stem extracts is generally linked to their TPC [17]. In this context, both mono-oligomeric and polymeric fractions of grape stem extracts are responsible for their total antioxidant activity; however, the polymeric fraction has been determined as the main contributor [22].

Leaving aside the difference in the polymeric procyanidin content, both extracts showed a similar trend in their general phenolic profile, although some differences in the amount of diverse individual phenolic compounds were recorded. Flavan-3-ols were the most abundant compounds in both extracts, and, to a lesser extent, phenolic acids, stilbenes, and flavanols. This phenolic composition is consistent with that reported by other authors, where an extended composition of flavan-3-ols has been observed in grape stem extracts, with catechin present in higher quantities than epicatechin. Moreover, phenolic acids, mainly gallic and caftaric acids, have been reported, although protocatechuic, *p*-hydroxybenzoic, vanillic, syringic, caffeic, or *p*-coumaric acids have also previously been found. In addition, *trans*-resveratrol, as well as diverse quercetin-related compounds, have previously been described in grape stem extracts [1,8]. Additionally, the characterization of the proanthocyanidin fraction was in agreement with the literature, particularly with Merlot’s proanthocyanidins [23].

### 3.2. In Vitro Gastrointestinal Digestion of Extracts

The antioxidant activity of the extracts throughout the digestion process followed the same trend as the TPC values, being clearly different in both extracts. Important reductions on the TPC values of HPE explained the observed loss of the antioxidant activity of this extract. On the other hand, LPE maintained its TPC values and its total antioxidant activity. Unfortunately, only Jara-Palacios et al. [7] and Ferreyra et al. [9] have studied the gastrointestinal digestion effect on the antioxidant activity of grape stem extracts, reporting reductions close to 30–50%, being in agreement with the HPE trend. In that regard, the bioaccessibility of phenolic compounds from food samples, and the corresponding antioxidant activity, can increase as a consequence of phenolic compound release from fibres [24]. However, for grape extracts, an antioxidant activity reduction due to a phenolic compound loss is commonly observed [5,22,25], although certain differences have been reported when different conditions were applied for phenolic extraction [7].

Previous studies have focused on the mono-oligomer fraction contribution to the antioxidant activity of wine-making by-product extracts during the digestion process. Gallic acid, catechin, and procyanidin B1 are mainly responsible for the antioxidant activity of grape stem extracts after digestion [7], agreeing with LPE since higher amounts of gallic acid, catechin, and procyanidin B1 bioaccessibility were observed in this extract compared to HPE. However, the mentioned study did not consider the contribution of the polymeric fraction to the final antioxidant activity of the digested extracts that represented the main antioxidant activity of HPE according to other studies [22]. Therefore, it seems plausible that the antioxidant activity trend is associated with the loss of polymeric procyanidins in the digestion process.

Regarding the individual phenolic compound bioaccessibility, the oral phase showed a marginal effect in HPE and LPE. However, important changes occurred during the stomach and intestinal phases, as reported previously in the literature [7,9]. Both extracts showed a reduction in flavan-3-ols and flavonols, whereas phenolic acids and stilbenes were more stable. It is important to point out that lower bioaccessibility was, in general, determined for individual compounds of HPE compared to LPE.

It has been described that the bioaccessibility of phenolic compounds may be modified because of their interaction with other components of the food matrix, such as proteins, complex carbohydrates, or lipids, although there are no references about the implication of ashes. However, there are not many studies aimed at demonstrating the influence of these different components on the bioaccessibility of individual phenolic compounds. In addition, the results shown in these studies were often opposite to those obtained in other studies because the interaction between phenolic compounds and the other components of the food matrix can sometimes depend specifically on the phenolic structure [13,14,15,16].

In general, there was a decrease in phenolic compounds after the gastric or intestinal steps in both extracts, in agreement with other studies [10,26]. In this context, Jara-Palacios et al. [7] determined a total degradation of gallic acid from grape stem extracts, whereas Ferreyra et al. [9] observed a bioaccessibility of 39% of these compounds. Tagliazucchi et al. [25] indicated a bioaccessibility close to 60% after pure gallic acid digestion, in agreement with the results observed for HPE (44%). Ferreyra et al. [9] reported low bioaccessibility for caftaric acid during grape stem extract digestion, whereas Garbetta et al. [27] indicated a high stability of this compound during grape skin digestion. 

The present study showed higher bioaccessibility rates for resveratrol from grape sources than other studies. Lee et al. [26] reported a 50% reduction of resveratrol bioaccessibility from grape extracts during gastrointestinal digestion, mainly due to the acid gastric conditions, whereas Ferreyra et al. [9] determined a complete loss of *trans*-resveratrol from grape cane extracts after gastric digestion. Moreover, this investigation also presented slightly higher bioaccessibility of flavonols than those obtained from grape stem extracts by other authors [7,9].

As mentioned above, very moderate increases in certain phenolic compounds were recorded after the digestion process. In that regard, the slightly higher content of 4-hydroxybenzoic acid and syringic acid in HPE could be attributable to their interaction with hemicelluloses as suggested by Wang et al. [28]. Moreover, Ortega et al. [29] found a clear influence of lipids on the enhanced bioaccessibility of phenolic acids and flavonoids that could be linked to the small rise of gallic acid, protocatechuic acid, *trans*-resveratrol, or dimer B_1_. Nevertheless, in some cases, these enhancements could also be ascribed to the degradation of phenolic structures [30], which would be consistent with the slight increase observed in this investigation and the procyanidin reduction. These results seem to indicate that a release of the phenolic compounds from the extract matrix did not occur or that the interactions with other components were weak.

Flavan-3-ols resulted in the lowest bioaccessible compounds. Several phenolic matrices, such as grape by-product extracts [31] or cocoa samples [32], have demonstrated that these compounds are extensively lost during the digestion process, where gastric and intestinal digestion are critical steps. Flavan-3-ols reductions during the digestion process may occur due to interactions with proteins from the food matrix; digestive proteins or structure modification of flavanols, such as dimerization of monomers; as well as adduct formations, conversions, and oxidative self-associations. Moreover, oligomeric procyanidins hydrolysis, such as dimer B_1_, into their constitutive monomers may occur during the stomach phase [33,34]. In the present study, an interaction between proteins of the extracts and flavan-3-ols [14,15] could be discarded, as similar drops in this family of compounds were recorded for both extracts, regardless of the different protein content of HPE and LPE. Therefore, flavan-3-ols mono- and oligomeric reductions might be mainly caused by specific interactions of these compounds with digestive enzymes, reducing the content of these compounds [35]. Oligomeric procyanidin precipitations have also been observed for grape seed extract digestion [31]. However, increases or reductions can be observed in other studies, depending on the analysed extract. In that respect, Ferreyra et al. [9] observed a marginal bioaccessibility of catechin for grape stem extracts. In contrast, important increases in catechin and epicatechin during gastric and intestinal digestion, but a total loss of dimer procyanidins, have been observed during grape stem extract digestion [7]. Intensive losses of flavan-3-ols were determined during the digestion of pure flavan-3-ol compounds, as well as for other grape or wine sources [10,11,25,27]. Duran-Castañeda et al. [36] observed different proanthocyanidin bioaccessibility results for various guava samples. These results supported the influence of the matrix effect.

On the other hand, the polymeric procyanidins resulted in a final bioaccessibility of 26% for the HPE. This is the first time that the bioaccessibility of the polymeric procyanidins from grape stem extracts was evaluated. Similar results were observed by Toro-Uribe et al. [32] who found cocoa procyanidin reductions from 60–80% after gastric digestion and less than 20% after intestinal digestion. Some studies have indicated a release of monomeric flavan-3-ol compounds as consequence of procyanidin degradations, in particular during the gastric step [10]; however, this phenomenon should be discarded in the present research since insignificant monomeric flavan-3-ols or phenolic acid (the main released compounds during procyanidin degradation) increments were determined for HPE. In agreement with mono- and oligomeric behaviour, these results were more consistent with an explanation based on the well-known interaction between polymeric procyanidins and the digestive enzymes than with the different protein content of both extracts [35,37]. The protein binding property of procyanidins was affected by the degree of polymerization, whereby an increase in the degree of polymerization was associated with enhanced protein precipitating capacity [38]. Abia and Fry [39] observed that 90–94% of the ingested carob proanthocyanidins remained undigested in the digested samples or feces in lab rats, where the compounds with a higher polymerization degree were precipitated with digestive enzymes. Gonthier et al. [40] observed neither parent compounds nor catechin derivatives in urine samples in rats fed with procyanidins, in contrast to those fed with catechin monomers, which excreted large amounts of catechin, suggesting procyanidin non-degradation during the digestion process. These previous investigations support the procyanidin precipitation suggested in this study.

It is important to highlight that, whereas TPC values were correlated with the antioxidant activity during the digestion process, the TPC was not consistent with the total phenolic compounds quantified by HPLC-PAD (as the sum of all the quantified compounds). A total bioaccessibility reduction of 71% was determined in HPE through HPLC-PAD quantification (close to TPC reductions), but lower bioaccessibility reductions were observed in TPC values (14%) of LPE compared to HPLC-PAD quantification, being 47%. Conversely, TPC reduction was close to the procyanidin reductions for both LPE and HPE. Changes in individual phenolic compounds could be a consequence of diverse events such as the interaction of the phenolic compounds with the digestive enzymes [11], phenolic-phenolic compound reactions [33], or possible transformations [9]. Structural changes in phenolic compounds, such as transformations or interactions among them, reduced their chromatographic identification and quantification, although they may still contribute to the TPC values (using the Folin Ciocalteau method) when digested samples are analysed. Therefore, the high decrease of the TPC values on HPE (as well as the low reductions in LPE) were mainly due to the loss of polymeric procyanidins. Conversely, the greater bioaccessibility of TPC observed for LPE was explained by its low amounts of polymeric procyanidins. Since antioxidant activity is a consequence of the TPC values during the digestion process [25], these data indicate that although the digestion process affected both mono-oligomeric and polymeric fractions, polymer reduction was the main contributor to the antioxidant activity decrease during this physiological process [41]. The antioxidant activity of the digested grape stem extracts has been associated with their bioaccessible compounds, such as epicatechin, naringin, gallic acid, procyanidin B_1_, or quercetin-3-*O*-glucoside [7,9]. However, these studies did not consider the contribution of the polymeric fraction to the antioxidant activity during gastrointestinal digestion, which was shown in this study for the first time.

The distinct bioaccessibility results of grape stem extracts found in our research, and in the literature, support the idea of a matrix effect on the phenolic compound bioaccessibility [5,9]. As a real implication of hemicellulose or proteins could be discarded to explain differences in the bioaccessibility of phenolics from HPE and LPE, other components, such as lipids or polymeric procyanidin, should be taken into account.

In connection with the latter, opposite results were also found in the literature for other phenolic grape matrixes [5,25]. For example, Gomes et al. [5] determined different bioaccessibility results for the digestion of whole grape samples compared to their isolated constitutive parts. Additionally, Lingua et al. [10] observed differences in the bioaccessibility results of whole grape digestion compared to wine samples derived from the same investigated grapes. This suggested an important influence of the polymeric fraction on the bioaccessibility and antioxidant activity of phenolic extracts during the digestion process. For example, Ferreyra et al. [9] observed that grape stem extracts showed, at their origin, higher amounts of catechin than those from grape canes, but 99% of catechin was reduced during gastrointestinal digestion of stem extracts compared to 70% of cane extracts. In contrast, Jara-Palacios et al. [7] found important increases in catechin and epicatechin in stem extracts during the gastrointestinal process. However, a study carried out with cocoa samples characterized by different fat content proposed the positive role of lipids to increase the bioaccessibility of phenolic acids and flavonoids, including oligomeric procyanidins [29], that agreed with the higher bioaccessibility of LPE phenolic compounds compared to HPE.

Therefore, the present research has highlighted the possible contribution of the polymeric procyanidin fraction, along with fat content, to the previous suggested matrix effect. These results explain the higher bioaccessibility of LPE (lower in TPC in origin, but with a higher content in lipids) compared to HPE, rich in polymeric procyanidins. These results show new evidence that suggests the need to conduct further research on grape stem bioaccessibility and bioavailability. Likewise, more studies are necessary to elucidate and explain the mechanisms involved in these interactions.

### 3.3. In Vitro Transepithelial Transport

Reduced increases of the antioxidant activity of the basolateral chamber occurred in both extracts after the digested in vitro transepithelial absorption. The antioxidant activity detected in the basolateral fraction of LPE was 2.6 times superior to the HPE basolateral fraction. The same trend was determined for the TPC values of the bioaccessible fractions.

Only some phenolic compounds were identified in the basolateral chambers, mainly flavan-3-ols, phenolic acids, and stilbenes. Phenolic acids—such as gallic, ferulic, and vanillic acid—have been reported previously as bioavailable compounds [10,11]. Deprez et al. [12] observed that catechin and procyanidin dimers may be transported by Caco-2 cells through a paracellular transport, in concordance with this study. However, the absorption of flavan-3-ol monomers is limited by continuous efflux to the apical side [42]. In addition, intestinal metabolization of phenolic compounds, such as flavan-3-ol monomers, may occur across the intestinal barrier, exporting derivative compounds from parent monomers to the basolateral side [43]. Nevertheless, the presence of flavan-3-ols such as catechin, epicatechin, and dimer B_1_ in urine and plasma samples of previous in vivo studies highlighted partial absorption of these compounds [44]. in vivo studies using lab rats fed with procyanidins did not observe urine catechin levels, whereas lab rats fed with monomeric catechin resulted in catechin urine excretion. These results support the idea that procyanidins are not degraded into monomers during the digestion process, in agreement with our research, since procyanidin losses were not associated with flavan-3-ol monomer increments during the digestion process [40].

The bioavailable phenolic compounds are responsible for the antioxidant activity of the basolateral chamber [10,24]. Thus, flavan-3-ols (mainly as catechin) and *trans*-resveratrol, as well as hydroxybenzoic acids detected in the bioavailable fractions, could be responsible for the remarkable higher antioxidant activity determined in LPE. These results are consistent with the presence of a greater amount of polymeric procyanidins in HPE since polymers have been reported to remain unabsorbed [40]. Pereira-Caro et al. [45] observed that rats fed with a red wine extract showed higher amounts of flavan-3-ol monomers in blood samples than those fed with a grape seed extract with a higher phenolic content, suggesting a lower bioavailability of the grape seed extract as a consequence of higher polymeric proanthocyanidins of the grape seed extract. Moreover, Rosa et al. [46] showed a significant decrease of ferulic acid in the presence of arabinoxylans, but, on the contrary, other studies reported a considerable increase of caffeoylquinic acids linked to a concentrate of water-soluble dietary fibres [47]. Ortega et al. [29] proposed a higher bioavailability of phenolic compounds linked to a higher fat content of cocoa liquor than cocoa powder, whereas other studies have detected interactions between oligomer procyanidins and lipid absorption [14].

It is important to point out that the digested HPE applied to transepithelial studies contained higher TPC values and antioxidant activity than the LPE digested. However, LPE resulted in higher bioavailability, showing greater TPC, antioxidant activity, and HPLC-PAD quantified phenolic compounds in the basolateral fraction. Higher amounts of phenolic compounds in digested extracts are associated with higher antioxidant bioavailability [24]. These results suggest a higher bioaccessibility and bioavailability in the LPE by its higher content of individual phenolic compounds after the digestion process rather than their total phenolic content, but also due to its higher content of fat. Hence, the extract matrix influence may be attributed not only to the bioaccessibility as was mentioned above but also to the final bioavailability. As a consequence, higher amounts of polymeric procyanidins could compromise the potential bioavailable antioxidant activity of the phenolic extracts, but the presence of other constituents, such as lipids, may exert a positive effect.

To our knowledge, this is the first time that the bioavailability of phenolic compounds from grape stem extracts has been studied, specifically the influence of the extract matrix. However, further investigations focused on the action mechanisms between procyanidins, lipids, digestive enzymes, and mono-oligomeric compounds are required to enhance the understanding of the impact of the polymeric procyanidins in the bioaccessibility and bioavailability of other phenolic compounds of phenolic extracts.

## 4. Materials and Methods

### 4.1. Chemicals and Reagents

Acetonitrile HPLC quality was supplied by Labscan (Dublin, Ireland), and formic acid HPLC quality was from Acros Organic (Geel, Belgium). Protocatechuic acid, vanillic acid, syringic acid, caffeic acid, 3-coumaric acid, ethyl gallate, 3,4′,5 trihydroxystilbene-3-*O*-d-glucoside (*trans*-piceid), (+)-catechin, (−)-epicatechin, epicatechin gallate, procyanidin B_1_, procyanidin B_2_, quercetin-3-*O*-galactoside (hyperoside), quercetin-3-*O*-rutinoside (rutin), quercetin-3-*O*-glucuronide, quercetin-3-*O*-glucoside, and quercetin dehydrate were purchased from Extrasynthèse (Genay, France). Glucose, xylose, phenol, gallic acid, 4-hydroxybenzoic acid, *trans*-caftaric acid, ellagic acid, protocatechuic aldehyde, *trans*-resveratrol, 6-hydroxy-2,5,7,8-tetramethylchromane-2-carboxylic acid (Trolox), potassium persulfate, 2,2´ azinobis(3-ethylbenzothiazoline-6-sulphonic acid) diammonium salt (ABTS), 2,2 diphenyl-1-picrylhydrazyl (DPPH), and 3-(4,5-dimethylthiazol-2-yl)-2,5-diphenyl tetrazolium bromide (MTT) were obtained from Sigma-Aldrich (Madrid, Spain). Disodium carbonate, Folin-Ciocalteau reagent, methanol, and ethanol were purchased from Panreac (Barcelona, Spain).

### 4.2. Plant Material

Red grapes of the Merlot variety (*Vitis vinifera* L. Merlot) were provided by Instituto Madrileño de Investigación y Desarrollo Rural, Agrario y Alimentario (IMIDRA, Madrid, Spain). After a manual de-stemming process, the resulting stem samples were cleaned with ultrapure water to remove most wastes. Then, water was immediately removed with soft laboratory paper, and stems were dried at 40 °C for 48 h in an air oven (Stuart S150, VWR, Barcelona, Spain). Dried material was ground and sieved (particle size ≤ 1 mm). The grape stem powder was stored in sealed plastic vials at −20 °C.

### 4.3. Pressurized Liquid Extraction (PLE)

The PLE conditions and process applied to the grape stem powder were based on a previous process developed by the research group, where the optimal extraction conditions for obtaining the highest antioxidant capacity and total phenolic content (TPC) values were determined [17]. Briefly, PLE extractions were carried out in an ASE 350 system from Dionex Corporation (Sunnyvale, CA, USA) equipped with a solvent controller unit. Extraction cells (11 mL of capacity) were filled with a mixture of 1 g of grape stem powder dispersed on 1 g of diatomaceous earth. Ethanol:water (30%) as a solvent, at 120 °C for 10 min, yielded HPE (high polymeric phenolic extract), whereas LPE (low polymeric phenolic extract) was obtained at 100% ethanol, 80 °C, and 6 min extraction time. Each extract was independently recovered in a glass vial, and ethanol was removed at 37 °C by vacuum rotary evaporation (IKA RV 10 control, IKA, Staufen, Germany). Next, the extracts were freeze-dried (Telstar Lyobeta 15 equipment; Telstar, Madrid, Spain) and stored at −20 °C in darkness until further analysis.

### 4.4. Composition Analysis

Protein (Kjeldahl nitrogen), ash, and fat content were analyzed according to the AOAC standarized methods 955.04/90, 942.05/90, and 920.39c, respectively [48].

For soluble sugar determination, 10 mg of extract was dissolved in ethanol:water or ethanol, respectively, for HPE or LPE. Quantification was carried out following the phenol-sulfuric method as described elsewhere [49]. Glucose was used as the standard.

Hemicelluloses were determined in HPE according to the phenol sulfuric procedure [50]. Previously, hemicelluloses were obtained from deffated HPE as detailed in the procedure of Lee et al. [51].

All analyses were performed in triplicate. Results are expressed as mean ± standard deviation.

### 4.5. HPLC-PAD Analysis of Phenolic Composition

Analysis of phenolic composition of extracts was conducted by HPLC-PAD using Agilent HPLC 1260 series equipment controlled by ChemStation software (Agilent, vers. 6.8) (Agilent Technologies Inc., Santa Clara, CA, USA). A C18 ACE RP18-AR column (150 mm × 4.6 mm, 3-µm particle size) (Symta, Madrid, Spain) protected by a guard column ACE 3 C18-AR (7 mm × 13 mm) was used for phenolic compound analysis. The chromatographic method, column temperature, sample injected volume, as well as sample preparation were carried out as described elsewhere [17]. The extracts and freeze-dried samples were dissolved in 1 mL of water:methanol (1:1) and then filtered by a 0.45-µm PVDF filter prior to analyses.

Quantification was carried out by external calibration curves with analytical standards (standard samples were prepared as decreased concentrations in 1 mL of water:methanol (1:1) and then filtered by a 0.45-µm PVDF filter), except for monogalloyl glucoside, which were identified and quantified according to gallic acid and *trans*-resveratrol standards, respectively. Hydroxybenzoic acids and flavan-3-ols were quantified at 280 nm, hydroxycinnamic acids and stilbenes at 320 nm, and flavonols at 360 nm. Calibration parameters—such as calibration curves, limit of detection, limit of quantification, and coefficient of determination—have been included in Table S1. Three different extracts were analysed, and all the analyses were conducted in triplicate.

### 4.6. Total Polymeric Procyanidin Content

The polymeric procyanidin fraction was isolated from PLE extracts employing the method described by Sun et al. [51]. Briefly, this method consists of two minicolumn assembly-line systems, on top of a tC18 Sep-Pack column followed by a C18 Sep-Pack (Merck, Darmstadt, Germany). Eighteen milligrams of extract samples (HPE or LPE) dissolved in 3 mL of water (pH adjusted to 7.0) were charged. The elution process was carried out with 10 mL of diluted phosphate buffer (1/8) followed by 5 mL of ultrapure water to remove phenolic acids. Next, 25 mL of ethyl acetate was added to elute catechin and oligomeric procyanidins. Finally, the polymeric procyanidin fraction was obtained by elution with 10 mL of methanol.

Total polymeric procyanidin content was analysed following the vanillin reaction method [52]. The obtained procyanidin fraction was dissolved in 5 mL of pure methanol. One milliliter of reagent A (1% vanillin in methanol) and 2.5 mL of reagent B (25% H_2_SO_4_ in methanol) were added to initiate the reaction. The absorbance was read at 500 nm after 10 min of reaction. The results were plotted in a pure catechin curve, being expressed as mg of catechin/g extract. The analyses were conducted in triplicate for the isolated procyanidin compounds obtained from 3 extract samples.

### 4.7. Determination of Mean Degree of Procyanidin Polymerization (mDP)

Flavan-3-ol procyanidins were isolated in a minicolumn assembly-line system (minicolumn cartridge C18 Sep-Pack and tC18 Sep-Pack from Waters, Milford, MA, USA). Next, degradation of isolated procyanidins was done by acid-catalyzed degradation using toluene-tiol. Quantification of degradation products and mDP were conducted by RP-HPLC-PAD [17]. The isolated procyanidin fractions were obtained from 3 distinct extracts, and the assays were carried out in triplicate.

### 4.8. Mono-Oligomeric and Polymeric Fraction Isolation

The mono-oligomeric and polymeric isolated fractions of the HPE were obtained according to Prodanov et al. [53] with some modifications. A special ultrafiltration dispositive consisting of an ultrafiltration membrane of 10 KDa coupled to a centrifugation tube (Merk, Millipore, Germany) was employed to isolate the low molecular phenolic compounds (mono-oligomeric fraction) from the polymeric fraction of HPE. A 12-mL sample (15 mg/mL of HPE dissolved in methanol:water (1:1)) was generated and placed into the membrane module. The sample was centrifuged (20 °C and 5000 rpm) for 4 h, allowing one to reach the complete filtration of the sample. As a consequence, the retained compounds remained on the ultrafiltration membrane selective side; meanwhile, the permeate passed through the membrane and was collected in the centrifugation tube. Both fractions were collected, and methanol was removed at 37 °C by vacuum rotary evaporation (IKA RV 10 control, IKA, Staufen, Germany). Finally, both fractions were freeze-dried (Telstar Lyobeta 15 equipment; Telstar, Madrid, Spain) and stored at −20 °C in darkness for further analysis. The isolation of the mono-oligomeric and procyanidin fractions was performed in triplicate from 3 individual extracts. Further analyses were carried out in triplicate.

### 4.9. Total Phenolic Content (TPC)

The Folin-Ciocalteau reagent method was used to determine the TPC values of the extracts [17]. The results were expressed as mg of gallic acid equivalents (GAE)/g extract. Analyses of 3 individual samples were conducted in triplicate.

### 4.10. Antioxidant Activity: DPPH Assay

The DPPH method was applied as described in Nieto et al. [17]. Trolox was used as the standard, and the antioxidant activity of the samples was expressed as the TEAC (Trolox Equivalent Antioxidant Capacity) (mmol Trolox equivalent/g extract). The assays were carried out in triplicate from 3 samples.

### 4.11. In Vitro Gastrointestinal Digestion

The digestion process was carried out following a previously reported protocol developed by our group [54], slightly modified. Briefly, both extracts were prepared at 30 mg/mL, and 5 mL of each dilution were mixed with 0.1 mL of α-amylase from human saliva type XIII-A (Sigma-Aldrich, Madrid, Spain) (9.3 mg in Cl_2_Ca 1 mM) and shaken for 2 min at 37 °C (oral phase). Stomach and intestinal phases were carried out using a titrator Tritinio Plus 877 (Methrom, Herisau, Switzerland). Thus, the oral phase sample was mixed with 25 mL of a gastric solution at pH 2 adjusted with 0.1 M HCl (127 mg of porcine pepsin from porcine mucosa 536 U/mg, Sigma Aldrich, Madrid, Spain) and shaken for 1 h at 37 °C (gastric phase). After gastric digestion, samples were adjusted to pH 7 with 1 M NaHCO_3_ prior to the pancreatic step. Next, a pancreatic-bile extract containing 9.3 mg of pancreatin (Sigma Aldrich, Madrid, Spain) and 115.7 mg of bile salts in 2.8 mL of 10 mM trizma-maleate buffer was added and incubated for 2 h at 37 °C (intestinal phase). At the end of the digestion process, the enzyme reaction was stopped immediately by cooling the samples in ice. Finally, samples were freeze-dried and kept at −20 °C until analysis. A digestion assay without extracts (control) was also performed. Three individual gastrointestinal digestion assays were carried out. Further analyses of the digestion steps were conducted in triplicate for each individual digestion assay.

### 4.12. In Vitro Transepithelial Transport

Caco-2 cells (American Type Culture Collection, ATCC, Manassas, VA, USA) were maintained in Dulbecco’s Modified Eagle’s Medium (DMEM) (Gibco, Barcelona, Spain) supplemented with 10% FBS, 100 U/mL penicillin, 100 mg/mL streptomycin, 1% nonessential amino acids, and 2 mM l-glutamine (Invitrogen, Barcelona, Spain) at 37 °C in an atmosphere containing 5% CO_2_. The cytotoxic effect of the digested extracts was tested on Caco-2 cells using MTT as described elsewhere [18].

For transport experiments, Caco-2 cells were seeded onto six-well Transwell^®^ plates (0.4-μm pore size, inserts of 24-mm diameter, Costar, Corning, Madrid, Spain) at a density of 3 × 105 cells per insert. The cells were maintained for 21 days, replacing culture medium every three days. The Caco-2 cell monolayers were used when transepithelial electrical resistance (TEER) (Evon World Precision Instruments, Sarasota, FL, USA) values were larger than 350 Ω cm^2^. Apical and basolateral compartments were washed once with PBS and then incubated with 1.5 mL and 1 mL of supplement DMEM without FBS, respectively, during 30 min prior to the experiments. Then, 100 µL of digested extracts were incorporated in the apical compartment and incubated for 6 h at 37 °C. TEER values were measured twice before and after experiments to monitor the integrity of the Caco-2 monolayer. Apical and basolateral collected samples were freeze-dried and stored at −20 °C prior to analysis. Three replicates of the digestion process were carried out, and phenolic compounds were analysed in triplicate. The transepithelial transport assays were conducted in triplicate for each digested sample.

### 4.13. Statistical Analysis

Experimental results were expressed as mean ± standard deviation (SD). Analysis of variance (one-way ANOVA) followed by Fisher’s least significant difference (LSD) test was used to discriminate among means at *p* < 0.05. A Pearson test was conducted to determine the correlation between experimental data. Statistical analyses were performed using Statgraphics v. Centurion XVI software for Windows (Statpoint Inc., Warrenton, VA, USA).

## 5. Conclusions

The present study shows that the matrix composition affects the bioaccessibility and bioavailability of phenolic compounds. Therefore, in order to obtain extracts with a high bioavailability of antioxidant phenolic compounds, a deep analysis of the main constituents of the extracts is mandatory. In this context, extraction of monomeric phenolic compounds would be preferable. The extraction conditions should be determined in order to obtain the highest amount of monomeric phenolic compounds while minimizing the extraction of polymeric procyanidins. In addition, the potential effect of other components of the extracts, such as lipids, on the bioaccessibility and bioavailability of phenolic compounds should be studied.

## Figures and Tables

**Table 1:** Composition of PLE grape stem extracts (% *w/w*).

Main Components	HPE	LPE
Proteins	14.3 ± 0.1 *	1.6 ± 0.0
Fat	13.9 ± 0.7	35.7 ± 1.9 *
Soluble sugars	24.8 ± 1.3	28.7 ± 0.9 *
Hemicelluloses	7.4 ± 0.6 *	-
Ash	15.8 ± 0.6 *	1.2 ± 0.1
Total phenolic content	18.5 ± 0.3 *	8.7 ± 0.0
Procyanidins	8.1 ± 0.7 *	1.1 ± 0.1

* Indicates significant differences between both samples using Fisher’s LSD test at *p* < 0.05. Data represent mean ± SD (*n* = 3).

**Table 2:** Individual phenolic composition of PLE grape stem extracts (mg compound/g extract).

Phenolic Compounds	HPE	LPE
**Hydroxybenzoic acids**		
Gallic acid	0.541 ± 0.029	0.726 ± 0.038 *
Protocatechuic acid	0.008 ± 0.000	0.017 ± 0.000 *
Monogalloyl glucoside	<LoQ	0.001 ± 0.000 *
4-Hydroxybenzoic acid	0.048 ± 0.001	0.173 ± 0.001 *
Vanillic acid	0.224 ± 0.010	0.505 ± 0.016 *
Syringic acid	0.202 ± 0.015	0.489 ± 0.020 *
Ethyl gallate	0.010 ± 0.001	0.015 ± 0.002
Ellagic acid	0.073 ± 0.004	0.130 ± 0.007 *
**Hydroxycinnamic acids**		
Caftaric acid	0.357 ± 0.003	0.183 ± 0.001 *
Caffeic acid	0.006 ± 0.000	0.014 ± 0.001 *
3-Coumaric acid	0.003 ± 0.000	<LoD
**Stilbenes**		
*trans*-Piceid	0.016 ± 0.000	0.049 ± 0.002 *
*trans*-Resveratrol	0.141 ± 0.003	1.215 ± 0.036 *
**Flavan-3-ols**		
Catechin	2.422 ± 0.034	6.662 ± 0.381 *
Epicatechin	1.293 ± 0.039	1.435 ± 0.073 *
Epicatechin gallate	0.245 ± 0.005	0.466 ± 0.009 *
Dimer B_1_	1.410 ± 0.034	1.028 ± 0.055 *
Dimer B_2_	0.349 ± 0.025	Co
**Flavonols**		
Quercetin-3-*O*-galactoside	0.047 ± 0.000	0.046 ± 0.001
Quercetin-3-*O*-rutinoside	0.029 ± 0.000	0.048 ± 0.005 *
Quercetin-3-*O*-glucuronide	1.425 ± 0.001	0.368 ± 0.012 *
Quercetin-3-*O*-glucoside	0.106 ± 0.004	0.167 ± 0.009 *
Quercetin	0.005 ± 0.000	0.019 ± 0.001 *
**Σ** **Phenolic compounds**	8.96 ± 0.008	13.76 ± 0.013 *

* Indicates significant differences between both samples using Fisher’s LSD test at *p* < 0.05. LoQ = limit of quantification. LoD = limit of detection. Co = coelution. Data represent mean ± SD (*n* = 9).

**Table 3:** Individual phenolic composition (monomers and oligomers) of HPE during a simulated digestion process (mg compound/g extract).

Phenolic Compounds	Initial	Oral	Stomach	Intestinal
**Hydroxybenzoic acids**				
Gallic acid	0.622 ± 0.003 ^b^	0.645 ± 0.001 ^a^	0.540 ± 0.002 ^c^	0.270 ± 0.024 ^d^
Protocatechuic acid	0.010 ± 0.000 ^a^	0.011 ± 0.000 ^a^	0.010 ± 0.000 ^a^	0.011 ± 0.002 ^a^
Monogalloyl glucoside	0.012 ± 0.001 ^a^	0.010 ± 0.000 ^b^	<LoD	<LoD
4-Hydroxybenzoic acid	0.058 ± 0.003 ^c^	0.060 ± 0.002 ^c^	0.068 ± 0.002 ^b^	0.074 ± 0.004 ^a^
Vanillic acid	0.209 ± 0.013 ^a^	0.201 ± 0.019 ^b^	Co	Co
Syringic acid	0.277 ± 0.004 ^b^	0.261 ± 0.012 ^c^	0.291 ± 0.002 ^a^	0.295 ± 0.008 ^a^
Ethyl gallate	0.010 ± 0.001 ^c^	0.010 ± 0.002 ^c^	0.014 ± 0.000 ^b^	0.021 ± 0.001 ^a^
Ellagic acid	0.077 ± 0.004 ^a^	0.072 ± 0.002 ^b^	0.037 ± 0.001 ^d^	0.046 ± 0.001 ^c^
**Hydroxycinnamic acids**				
Caftaric acid	0.165 ± 0.002 ^b^	0.173 ± 0.000 ^a^	0.148 ± 0.001 ^c^	0.130 ± 0.005 ^d^
Caffeic acid	0.006 ± 0.000 ^b^	0.006 ± 0.000 ^b^	0.011 ± 0.000 ^a^	Co
3-Coumaric acid	0.003 ± 0.000 ^a^	0.003 ± 0.000 ^a^	<LoD	<LoD
**Stilbenes**				
*trans*-Piceid	0.014 ± 0.000 ^a^	0.015 ± 0.000 ^a^	0.014 ± 0.000 ^b^	0.014 ± 0.000 ^b^
*trans*-Resveratrol	0.263 ± 0.004 ^a^	0.273 ± 0.001 ^a^	0.218 ± 0.001 ^b^	0.199 ± 0.015 ^c^
**Flavan-3-ols**				
Catechin	2.749 ± 0.010 ^a^	2.755 ± 0.169 ^a^	1.885 ± 0.002 ^b^	0.368 ± 0.037 ^c^
Epicatechin	0.851 ± 0.003 ^a^	0.892 ± 0.023 ^a^	0.683 ± 0.001 ^b^	0.052 ± 0.029 ^c^
Epicatechin gallate	0.230 ± 0.003 ^a^	0.234 ± 0.004 ^a^	<LoD	<LoD
Dimer B_1_	1.562 ± 0.004 ^a^	1.577 ± 0.055 ^a^	1.434 ± 0.004 ^b^	0.248 ± 0.044 ^c^
Dimer B_2_	0.508 ± 0.027 ^a^	0.521 ± 0.010 ^a^	0.293 ± 0.007 ^b^	Co
**Flavonols**				
Quercetin-3-*O*-galactoside	0.018 ± 0.001 ^a^	0.018 ± 0.001 ^a^	0.005 ± 0.001 ^c^	0.009 ± 0.003 ^b^
Quercetin-3-*O*-rutinoside	0.019 ± 0.001 ^a^	0.018 ± 0.001 ^a^	0.015 ± 0.001 ^b^	0.014 ± 0.002 ^b^
Quercetin-3-*O*-glucuronide	0.784 ± 0.010 ^a^	0.797 ± 0.006 ^a^	0.555 ± 0.002 ^c^	0.629 ± 0.051 ^b^
Quercetin-3-*O*-glucoside	0.106 ± 0.004 ^a^	0.110 ± 0.004 ^a^	0.082 ± 0.002 ^b^	0.073 ± 0.003 ^c^
Quercetin	0.004 ± 0.000 ^a^	0.005 ± 0.000 ^a^	0.001 ± 0.000 ^b^	< LoD
**Σ** **Phenolic compounds**	8.557 ± 0.006 ^a^	8.670 ± 0.013 ^a^	6.287 ± 0.001 ^b^	2.454 ± 0.010 ^c^

^a,b,c,d^ Different letters indicate significant differences in a compound content between digestion steps using Fisher’s LSD test at *p* < 0.05. LoD = limit of detection. Co = coelution. Data represent mean ± SD (*n* = 9).

**Table 4:** Individual phenolic composition (monomers and oligomers) of LPE during a simulated digestion process (mg compound/g extract).

Phenolic Compounds	Initial	Oral	Stomach	Intestinal
**Hydroxybenzoic acids**				
Gallic acid	0.726 ± 0.038 ^a,b^	0.735 ± 0.042 ^a,b^	0.788 ± 0.033 ^a^	0.671 ± 0.047 ^b^
Protocatechuic acid	0.017 ± 0.002 ^a^	0.017 ± 0.000 ^a^	0.019 ± 0.000 ^a^	0.017 ± 0.002 ^a^
Monogalloyl glucoside	0.005 ± 0.002 ^a^	0.005 ± 0.000 ^a^	< LoD	< LoD
4-Hydroxybenzoic acid	0.173 ± 0.011 ^b^	0.182 ± 0.009 ^a,b^	0.168 ± 0.011 ^b^	0.194 ± 0.004 ^a^
Vanillic acid	0.505 ± 0.016 ^a^	0.493 ± 0.012 ^a^	Co	Co
Syringic acid	0.489 ± 0.020 ^b^	0.535 ± 0.018 ^a^	0.507 ± 0.002 ^a,b^	0.485 ± 0.029 ^b^
Ethyl gallate	0.015 ± 0.002 ^c^	0.014 ± 0.001 ^c^	0.019 ± 0.000 ^b^	0.021 ± 0.000 ^a^
Ellagic acid	0.131 ± 0.007 ^a^	0.130 ± 0.009 ^a^	0.012 ± 0.000 ^c^	0.035 ± 0.005 ^b^
**Hydroxycinnamic acids**				
Caftaric acid	0.018 ± 0.001 ^a^	0.018 ± 0.001 ^a^	0.019 ± 0.000 ^a^	0.018 ± 0.002 ^a^
Caffeic acid	0.015 ± 0.003 ^a^	0.018 ± 0.001 ^a^	0.012 ± 0.000 ^b^	0.011 ± 0.002 ^b^
3-Coumaric acid	<LoD	<LoD	<LoD	<LoD
**Stilbenes**				
*trans*-Piceid	0.049 ± 0.002 ^a^	0.050 ± 0.003 ^a^	0.047 ± 0.000 ^a^	0.041 ± 0.002 ^b^
*trans*-Resveratrol	1.215 ± 0.036 ^b^	1.366 ± 0.121 ^a,b^	1.415 ± 0.035 ^a^	1.499 ± 0.116 ^a^
**Flavan-3-ols**				
Catechin	6.662 ± 0.380 ^a^	6.954 ± 0.383 ^a^	3.754 ± 0.004 ^b^	2.102 ± 0.046 ^c^
Epicatechin	1.435 ± 0.073 ^a^	1.478 ± 0.008 ^a^	1.200 ± 0.008 ^b^	0.718 ± 0.060 ^c^
Epicatechin gallate	0.466 ± 0.009 ^a^	0.490 ± 0.022 ^a^	<LoQ	0.064 ± 0.003 ^b^
Dimer B_1_	1.028 ± 0.055 ^b^	0.891 ± 0.076 ^b^	1.201 ± 0.007 ^a^	0.720 ± 0.095 ^c^
Dimer B_2_	Co	Co	Co	Co
**Flavonols**				
Quercetin-3-*O*-galactoside	0.046 ± 0.001 ^a^	0.046 ± 0.004 ^a^	0.018 ± 0.000 ^c^	0.026 ± 0.001 ^b^
Quercetin-3-*O*-rutinoside	0.048 ± 0.005 ^a^	0.047 ± 0.008 ^a^	0.027 ± 0.001 ^c^	0.036 ± 0.000 ^b^
Quercetin-3-*O*-glucuronide	0.386 ± 0.012 ^a^	0.402 ± 0.002 ^a^	0.243 ± 0.002 ^c^	0.287 ± 0.013 ^b^
Quercetin-3-*O*-glucoside	0.167 ± 0.009 ^a^	0.163 ± 0.006 ^a^	0.167 ± 0.001 ^a^	0.163 ± 0.005 ^a^
Quercetin	0.009 ± 0.000 ^a^	0.010 ± 0.002 ^a^	0.001 ± 0.000 ^c^	0.003 ± 0.000 ^b^
**Σ Phenolic compounds**	13.604 ± 0.030 ^a^	14.046 ± 0.032 ^a^	9.605 ± 0.004 ^b^	7.111 ± 0.019 ^c^

^a,b,c^ Different letters indicate significant differences in a compound content between digestion steps using Fisher’s LSD test at *p* < 0.05. LoQ = limit of quantification. LoD = limit of detection. Co = coelute. Data represent mean ± SD (*n* = 9).

**Table 5:** Phenolic compounds recovered from the apical (unabsorbed) and basolateral (bioavailable) compartments during the uptake experiment conducted on Caco-2 cell monolayers (mg compound/g of extract).

Extracts	HPE			LPE		
Phenolic Compounds	Digested	Apical	Basolateral	Digested	Apical	Basolateral
**Hydroxybenzoic acids**						
Gallic acid	0.270 ± 0.024 ^a^	0.217 ± 0.002 ^b^	0.064 ± 0.008 ^c^	0.671 ± 0.047 ^a^	0.279 ± 0.001 ^b^	0.132 ± 0.008 ^c^
Protocatechuic acid	0.011 ± 0.002 ^a^	0.008 ± 0.000 ^b^	0.002 ± 0.000 ^c^	0.017 ± 0.002 ^a^	Co	0.011 ± 0.001 ^b^
Monogalloyl glucoside	<LoD	<LoD	<LoD	<LoD	<LoD	<LoD
4-Hydroxybenzoic acid	0.074 ± 0.004 ^a^	0.049 ± 0.012 ^b^	0.028 ± 0.004 ^c^	0.194 ± 0.004 ^a^	0.082 ± 0.003 ^c^	0.122 ± 0.012 ^b^
Vanillic acid	Co	<LoD	<LoD	Co	Co	0.301 ± 0.017
Syringic acid	0.295 ± 0.008 ^a^	0.212 ± 0.006 ^b^	0.076 ± 0.002 ^c^	0.485 ± 0.029 ^a^	0.345 ± 0.026 ^b^	0.218 ± 0.013 ^c^
Ethyl gallate	0.021 ± 0.000 ^a^	0.009 ± 0.002 ^b^	<LoD	0.021 ± 0.000 ^a^	0.010 ± 0.003 ^b^	0.004 ± 0.000 ^c^
Ellagic acid	0.046 ± 0.001 ^a^	0.032 ± 0.002 ^b^	<LoD	0.035 ± 0.005 ^a^	0.013 ± 0.000 ^b^	<LoD
**Hydroxycinnamic acids**						
Caftaric acid	0.130 ± 0.005 ^a^	0.071 ± 0.003 ^b^	0.017 ± 0.001 ^c^	0.018 ± 0.002	<LoQ	<LoQ
Caffeic acid	Co	<LoD	<LoD	0.011 ± 0.002 ^a^	<LoQ	0.12 ± 0.001 ^b^
3-Coumaric acid	<LoD	<LoD	<LoD	<LoD	<LoD	<LoD
**Stilbenes**						
*trans*-Piceid	0.014 ± 0.000 ^a^	0.013 ± 0.000 ^b^	0.003 ± 0.000 ^c^	0.041 ± 0.002 ^a^	0.030 ± 0.000 ^b^	0.011 ± 0.001 ^c^
*trans*-Resveratrol	0.199 ± 0.015 ^a^	0.006 ± 0.000 ^c^	0.019 ± 0.000 ^b^	1.499 ± 0.116 ^a^	0.211 ± 0.005 ^c^	0.325 ± 0.010 ^b^
**Flavan-3-ols**						
Catechin	0.368 ± 0.037 ^a^	Co	0.215 ± 0.001 ^b^	2.102 ± 0.046 ^a^	1.249 ± 0.064 ^b^	0.688 ± 0.006 ^c^
Epicatechin	0.052 ± 0.029	<LoD	<LoD	0.718 ± 0.060 ^a^	0.330 ± 0.060 ^b^	0.174 ± 0.010 ^c^
Epicatechin gallate	<LoD	<LoD	<LoD	0.064 ± 0.003	<LoD	<LoD
Dimer B_1_	0.248 ± 0.044 ^a^	0.113 ± 0.008 ^b^	<LoD	0.720 ± 0.095 ^a^	0.127 ± 0.019 ^b^	0.083 ± 0.002 ^c^
Dimer B_2_	Co	<LoD	<LoD	Co	<LoD	<LoD
**Flavonols**						
Quercetin-3-*O*-galactoside	0.009 ± 0.003 ^a^	0.009 ± 0.000 ^a^	<LoD	0.026 ± 0.001 ^a^	0.018 ± 0.001 ^b^	0.002 ± 0.000 ^c^
Quercetin-3-*O*-rutinoside	0.014 ± 0.002 ^a^	0.013 ± 0.000 ^a^	<LoD	0.036 ± 0.000 ^a^	0.025 ± 0.001 ^b^	<LoD
Quercetin-3-*O*-glucuronide	0.629 ± 0.051 ^a^	0.507 ± 0.004 ^b^	0.040 ± 0.001 ^c^	0.287 ± 0.013 ^a^	0.238 ± 0.005 ^b^	0.056 ± 0.008 ^c^
Quercetin-3-*O*-glucoside	0.073 ± 0.003 ^a^	0.038 ± 0.000 ^b^	<LoQ	0.163 ± 0.005 ^a^	0.061 ± 0.003 ^b^	0.047 ± 0.002 ^c^
Quercetin	<LoD	<LoD	<LoD	0.003 ± 0.000 ^a^	0.001 ± 0.000 ^b^	<LoQ
**Σ** **Phenolic compounds**	2.454 ± 0.010 ^a^	1.434 ± 0.001 ^b^	0.536 ± 0.001 ^c^	7.111 ± 0.017 ^a^	3.087 ± 0.008 ^b^	2.274 ± 0.004 ^c^

^a,b,c^ Different letters indicate significant differences during the intestinal absorption process using Fisher’s LSD test at *p* < 0.05. LoQ = limit of quantification. LoD = limit of detection. Co = coelute. Data represent mean ± SD (*n* = 9).

## Data Availability

Data is included in the article.

## Supplementary Materials

The following supporting information can be downloaded at: https://www.mdpi.com/article/10.3390/molecules28062461/s1, **Table S1:** Chemical characterization of the isolated procyanidin fraction. **Table S2:** Calibration curves, LoD, LoQ and coefficient of determination of the reference standards. **Figure S1:** Correlation between mono-oligomeric fraction (grey) and procyanidin fraction (black) content and antioxidant activity during gastrointestinal digestion of the HPE extract.

## Sample Availability

Not available.
