# Supplementary material for: Implication of the Polymeric Phenolic Fraction and Matrix Effect on the Antioxidant Activity, Bioaccessibility, and Bioavailability of Grape Stem Extracts
Source: Molecules. 2023 Mar 8;28(6):2461. doi: 10.3390/molecules28062461 (PMC10051231; doi:10.3390/molecules28062461)
Supplement: Supplementary file 1 [file molecules-28-02461-s001.zip › molecules-2102246-supplementary.pdf]

Implication of the Polymeric Phenolic Fraction and Matrix Effect on the Antioxidant Activity, Bioaccessibility, and Bioavailability of Grape Stem Extracts

Juan Antonio Nieto, Irene Fernández-Jalao, María de las Nieves Siles-Sánchez, Susana Santoyo and Laura Jaime *

Table S1. Chemical characterization of the isolated procyanidin fraction.

Extracts	Catechin (%)	Epicatechin (%)	Epicatechin gallate (%)	Epigallocatechin (%)	mDP
**HPE**	14.59 ± 0.62	72.33 ± 0.49	12.40 ± 0.14	0.68 ± 0.11	12.22 ± 0.68
**LPE**	16.58 ± 0.98	67.29 ± 2.53	15.59 ± 1.66	0.55 ± 0.05	12.18 ± 0.97

mDP = medium degree of polymerization.

Table S2. Calibration curves, LoD, LoQ and coefficient of determination of the reference standards.

Phenolic compounds	Equation	LoD (mg/L)	LoQ (mg/L)	R^2
Gallic acid	y = 109324x – 9.487	0.393	1.190	0.9998
Protocatechuic acid	y = 608845x – 2.291	0.014	0.043	1.0000
4-Hydroxybenzoic acid	y = 65860x – 2.902	0.198	0.600	0.9994
Vanillic acid	y = 12805x – 0.823	0.546	1.655	0.9996
Syringic acid	y = 41846x – 0.4823	0.171	0.519	0.9999
Caftaric acid	y = 107084x – 2.0433	0.238	1.071	0.9996
Caffeic acid	y = 245503x – 5.766	0.024	0.127	0.9999
3-Coumaric acid	y = 69330x – 2.207	0.045	0.160	0.9997
Ethyl gallate	y = 124694x – 3.274	0.069	0.209	0.9998
Ellagic acid	y = 71898x – 3.854	0.197	0.882	0.9996
trans-Piceid	y = 240019x – 2.9723	0.012	0.073	0.9999
trans-Resveratrol	y = 255118x – 9.0471	0.056	0.308	0.9995
Catechin	y = 14394x – 31.0908	2.515	7.622	0.9998
Epicatechin	y = 10481x + 11.8033	2.801	8.488	0.9995
Epicatechin gallate	y = 37081x – 23.8862	1.930	5.849	0.9995
Dimer B_1	y = 14342x – 15.8623	2.208	6.690	0.9991
Dimer B_2	y = 10007x + 8.2927	2.140	6.486	0.9994
Quercetin-3-*O*-galactoside	y = 65620x – 2.4850	0.135	0.608	0.9995
Quercetin-3-*O*-rutinoside	y = 48776x – 12.897	0.108	0.381	0.9998
Quercetin-3-*O*-glucuronide	y = 58579x – 3.382	0.158	0.558	0.9996
Quercetin-3-*O*-glucoside	y = 86103x – 29.5653	0.096	0.422	0.9997
Quercetin	y = 184585x + 4.1978	0.035	0.190	0.9995

LoQ = limit of quantification. LoD = limit of detection.

HPLC-PAD calibration parameters

Calibration curves: Linearity was obtained from at least five different concentrations of standard solutions in triplicate.

LODs and LOQs: Limits of detection (LODs) and quantification (LOQs) were calculated based on the standard deviation of the regression line and its slope express as:

LoD = 3.3σ/S and LoQ = 10σ/S

Where σ = standard deviation of the regression line

S = the slope of the regression line

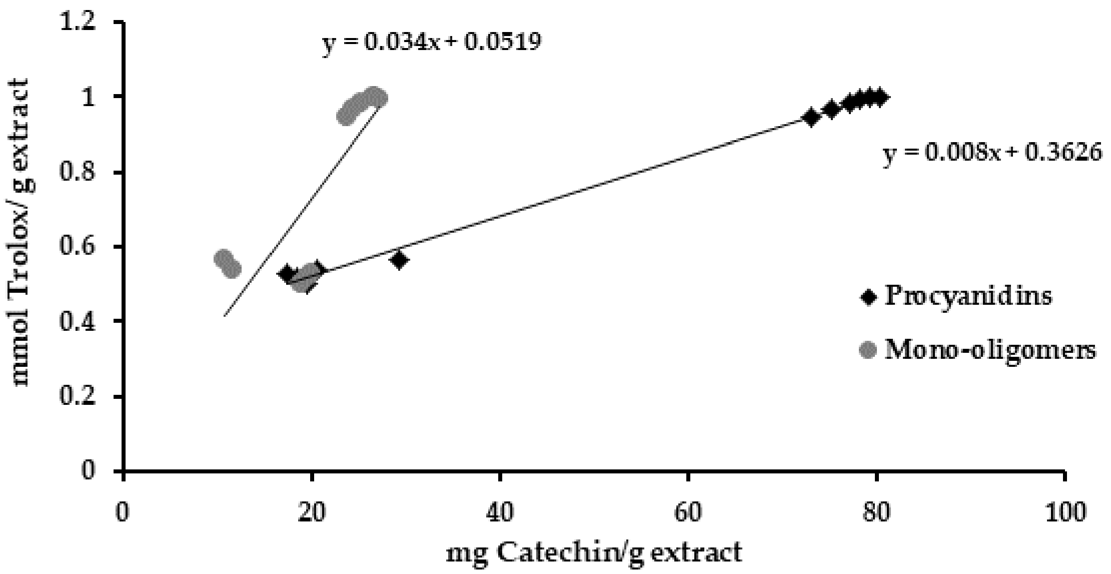

Figure S1. Correlation between mono-oligomeric fraction (grey) and procyanidin fraction (black) content and antioxidant activity during gastrointestinal digestion of the HPE extract.
